# Early derivation of IgM memory cells and bone marrow plasmablasts

**DOI:** 10.1371/journal.pone.0178853

**Published:** 2017-06-02

**Authors:** Amber M. Papillion, Kevin J. Kenderes, Jennifer L. Yates, Gary M. Winslow

**Affiliations:** 1 Department of Microbiology and Immunology, Upstate Medical University, Syracuse, New York, United States of America; 2 Wadsworth Center, and University at Albany, Albany, New York, United States of America; New York State Department of Health, UNITED STATES

## Abstract

IgM memory cells are recognized as an important component of B cell memory in mice and humans. Our studies of B cells elicited in response to ehrlichial infection identified a population of CD11c-positive IgM memory cells, and an IgM bone marrow antibody-secreting cell population. The origin of these cells was unknown, although an early T-independent spleen CD11c- and T-bet-positive IgM plasmablast population precedes both, suggesting a linear relationship. A majority of the IgM memory cells detected after day 30 post-infection, also T-bet-positive, had undergone somatic hypermutation, indicating they expressed activation-induced cytidine deaminase (AID). Therefore, to identify early AID-expressing precursor B cells, we infected an AID-regulated tamoxifen-inducible Cre-recombinase-EYFP reporter strain. Tamoxifen administration led to the labeling of both IgM memory cells and bone marrow ASCs on day 30 and later post-infection. High frequencies of labeled cells were identified on day 30 post-infection, following tamoxifen administration on day 10 post-infection, although IgM memory cells were marked when tamoxifen was administered as early as day 4 post-infection. Transcription of *Aicda* in the early plasmablasts was not detected in the absence of CD4 T cells, but occurred independently of TLR signaling. Unlike the IgM memory cells, the bone marrow IgM ASCs were elicited independent of T cell help. Moreover, *Aicda* was constitutively expressed in IgM memory cells, but not in bone marrow ASCs. These studies demonstrate that two distinct long-term IgM-positive B cell populations are generated early in response to infection, but are maintained via separate mechanisms.

## Introduction

Memory B cells, in addition to long-lived plasma cells, provide a major component of immunological memory [1, 2]. Although it has often been assumed that B cell memory is harbored in high-affinity class-switched immunoglobulin (swIg) B cells, it has become increasingly apparent that, as for T cells, the memory B cell compartment is diverse, and several different memory subsets exist [3–5]. There is considerable phenotypic heterogeneity, i.e., varying surface markers and Ig expression, within populations of hapten-elicited memory cells [6], differences which may reflect different kinds of memory cell functions [7]. Moreover, several studies have revealed that unswitched murine IgM B cells harbored a significant component of humoral memory [8–11]. IgM memory cells have been characterized in studies of murine memory responses following immunization, and similar cells are found in humans [12, 13]. IgM memory cells constitute a novel and important subset of long-lived memory B cells that may provide immunity to variant pathogens not recognized by classical high-affinity swIg memory B cells [14, 15].

In addition to memory B cells, bone marrow plasma cells constitutively produce class-switched antibodies that mediate long-term immunity [16–18]. Switched plasma cells have long been considered to be the major source of long-term antibodies, although several studies have described long-term bone marrow IgM antibody-secreting cells (ASCs; [19, 20]). T cell-independent (TI) antigens can induce bone marrow IgM ASCs, although it has often been considered that this response is short-lived [21, 22]. Our previous studies have indicated, however, that unswitched B cells and IgM can play an important role in long-term immunity to pathogens [20, 23].

Our studies of B cells during infection have utilized a mouse model of ehrlichiosis caused by the intracellular monocytotropic bacterial pathogen, *Ehrlichia muris*. We first identified a TI CD11c-positive splenic plasmablast population present on and about day 10 post-infection that is responsible for the initial production of antigen-specific IgM during infection [24]. This day 10 CD11c-positive splenic plasmablast population produces pathogen-specific polyreactive IgM, is not found in GCs, and is generated in the absence of CD4 T cell help [24, 25]. A second population of splenic CD19- and CD11c-positive B cells is elicited within 3–4 weeks post-infection, and is detected at relatively high frequencies for at least as long as one year post-infection [23]. We have demonstrated that these CD19/CD11c-positive B cells are IgM memory B cells, based on a number of definitive criteria, including their expression of the integrins CD11c and CD11b, as well as many other markers previously identified on memory B cells, such as CD73, PD-L2, CD80, and CD38 [23, 26]. Moreover, the cells are largely quiescent, do not reside in GCs, have undergone limited somatic mutation, and are responsible for anamnestic, memory responses following antigen challenge [23]. The latter studies indicated that the CD11c-positive IgM memory population, which were only detected in infected mice, was composed, at least in part, of antigen-specific B cells. In addition to our work, studies of B cell responses in other experimental animal models and humans have identified what are likely related memory cells [15, 27, 28]. The spleen is a major reservoir of memory B cells, including IgM memory cells (both IgD-negative and -positive) in humans [29–31]. Human IgM memory cells are elicited in response to *Streptococcus pneumoniae* infection [30], malaria infection [11], and following tetanus immunization [32].

The early CD11c-positive plasmablasts and IgM memory cells that we have described also express the transcriptional factor T-bet. B cells that express either CD11c, T-bet, or both molecules, have been identified in both human and animals in response to immunization, infections, and in autoimmunity [33–38]. The identification of CD11c-positive T-bet+ cells in aged autoimmune patients led to their description as Age-Related B cells (ABCs; [36, 37, 39]), although CD11c-positive T-bet+ B cells are now known to function in many different immunological contexts. Whether CD11c and T-bet expression define a monolithic B cell population, or a number of related but functionally distinct B cell subsets, is currently unresolved. Our studies have indicated that CD11c- and T-bet-positive B cells include both early TI plasmablasts and IgM memory cells [23, 24]. The derivation of and relationship between these two subsets remained unresolved in our previous studies, however.

We have also described a third non-canonical population of IgM T-bet-positive ASCs that arises in the bone marrow of infected mice after peak infection [20]. These B cells express CD138, CD93, and CD44, but are CD11c-negative, and are responsible for the production of protective long-term IgM [20]. Thus, ehrlichial infection generates two diverse populations of long-lived IgM-positive B cells, in the spleen and bone marrow, respectively. The phenotypic similarity between these two populations, as well as the observation that the day 10 TI CD11c-positive plasmablasts precede both the IgM memory cells and bone marrow ASCs, suggested that the day 10 CD11c-positive B cells, or a yet unidentified population, are the precursors to one or both long-term populations. Here we demonstrate that both long-term IgM populations are derived from B cells elicited early following infection, at the time of the peak CD11c-positive plasmablast response. Moreover, because the bone marrow ASCs and IgM memory cells differ in their requirement for CD4 T cell help, we suggest that B cell fate is determined by the availability of signals from T_fh_ cells that are also present in abundance in the spleen during infection. Finally, we show that although the day 10 CD11c-positive plasmablasts are generated independently of CD4 T cells, they nevertheless require CD4 T cells, likely T_fh_ cells, for the induction of *Aicda* mRNA and for AID expression. These findings add to our understanding of the generation of non-canonical CD11c- and T-bet-positive B cell memory and effector cell subsets in the context of an intracellular bacterial infection.

## Materials and methods

### Ethics statement

Our study was approved by the IACUC committee at SUNY Upstate Medical University (CHU 311), and is in accordance with the guidelines established in the Guide for the Care and Use of Laboratory Animals by the National Institutes of Health.

### Mice

Sex-matched C57BL/6, B6.Cg-Gt(Rosa)26Sor^tm3(CAG-EYFP)Hze^/J and MHC class II (MHCII)-deficient (B6.129S2-H2^dlAb1-Ea/J^) mice were obtained from The Jackson Laboratory (Bar Harbor, ME). The AID-Cre-ER^T2^ mice were generously provided by Dr. Jean-Claude Weill, ISERM, Paris, France. The UNC 93b deficient mice were provided by Dr. Ann Marshak-Rothstein, University of Massachusetts Medical School. All mice were bred and maintained under microisolator conditions under light/dark conditions in cages with bedding (up to five mice per cage), at Upstate Medical University, in accordance with institutional guidelines for animal welfare. The mice were maintained continuously under microisolator conditions. All experimental mice were between six and eight weeks of age, and within normal ranges of weight at the time of infection. Each mouse was considered to be an experimental unit. All mice were euthanized using CO_2_. When anesthesia was necessary, the mice were treated with isoflurane and were monitored following recovery from anesthesia.

### Genotyping

Mouse genomic DNA was extracted from tail tissue using hot sodium hydroxide, as previously described [40]. PCR was performed using the following oligonucleotide primers: eYFP (internal positive control forward 5’-CAAATGTTGCTTGTCTGGTG-3’; internal positive control reverse 5’-TCAGTGGGAATTAGTCATGCC-3’; transgene forward 5’-GGGACCATGAAGCTGCTGCCG-3’; transgene reverse 5’-GGCATTAAAGCAGCGTATCG-3’; the reactions yielded 625 and 200 bp products from the transgene and internal positive control alleles, respectively).

### Infections and treatments

Mice were infected i.p. with 5x10^4^ copies of *E*. *muris*, as previously described [41]. *E*. *muris* causes a non-fatal infection in immunocompetent mice. None of the mice in this study showed signs of serve illness following infection, nor did any of the mice die due to infection. Mice were monitored daily. CD40L blockade was performed by administration of 200 μg of the mAb MR-1 (anti-CD40) on days 8, 10, and 12 post-infection; an irrelevant isotype-matched antibody (clone 2A3) was used a control. Tamoxifen was dissolved in peanut oil at a concentration of 20mg/ml, and 0.5 ml was administered via oral gavage. To validate the effectiveness of the CD40L blockade, NP-BSA-immunized C57BL/6 mice were administered either 200 μg of 2A3 or MR-1 on days 4, 8, and 12 post-immunization.

### Cell transfer studies

To obtain B cells for cell transfer studies, spleens from three naïve AID-Cre-ER^T2^ x B6.Cg-Gt(Rosa)26Sor^tm3(CAG-EYFP)Hze^/J F1 mice were harvested and pooled. T cells were depleted by magnetic bead negative selection, using a CD90.2 T cell enrichment kit (Stem Cell Technologies). Naïve C57BL/6 and MHCII-deficient mice were injected with 7.7 x 10^6^ naïve AID-Cre-ER^T2^ x B6.Cg-Gt(Rosa)26Sor^tm3(CAG-EYFP)Hze^/J F1 B cells i.v., and the mice were infected i.p. with *E*. *muris*. The MHCII-deficient recipient mice were treated on day 0 and 3 post-infection with 200 μg GK1.5 antibody, i.p., to deplete any co-transferred CD4 T cells. The mice were then infected with *E*. *muris*, and were administered 10 mg tamoxifen oral gavage on days 7 and 10 post-infection.

### Flow cytometry and antibodies

Spleen and bone marrow cells were disaggregated using a 70μm cell strainer (BD Biosciences), and erythrocytes removed by hypotonic lysis, using ammonium chloride. Cells were treated with anti-CD16/32 (2.4G2) prior to incubation with the following antibodies: anti-IgM (clone II/41), eBioscience, San Diego, CA), IgM (R6-60.2 BD Biosciences), B220 (RA3-6B2, BD), B220 (RA3-6B2; eBioscience), CD138 (281–2), and CD11c (HL3; BD Biosciences). The cells were stained at 4°C for 20 min, washed, and analyzed without fixation for marker expression. Data were acquired on a BD Fortessa flow cytometer with Diva software (BD Biosciences), and were analyzed using FlowJo software (Tree Star, Inc.).

### RT-PCR

RNA was extracted from sorted CD11c+ B220+ using TRIzol, following the manufacturer’s protocol (Life Technologies). cDNA was generated using a Tetro cDNA Synthesis Kit (Bioline). RT-qPCR was performed using a BioRad T100 Thermo Cycler; transcripts were normalized to β-actin (Life Technologies probe Mm00607939_s1) expression. *Aicda* mRNA was detected using a primer-probe set specified by Life Technologies (Mm01184115_m1). To generate a positive control reagent, murine *Aicda* was amplified by PCR using Phusion High-Fidelity DNA Polymerase (New England BioLabs), using the following oligonucleotide primers: CTACCTCTGCTACGTGGTGAA (forward), GCTGAGGTTAGGGTTCCATCT (reverse). The amplicon was cloned using a TOPO TA cloning kit (Life Technologies).

### TLR9 stimulation

Spleen cells were obtained from C57BL/6 and UNC93b-deficient mice after T-cell-depletion and B cell enrichment. The cells were labeled with CFSE and cultured with or without CpG (ODN1826). Cell stimulation was measured by monitoring dilution of CFSE after three days in culture.

### Statistical analyses

Statistical analyses were performed using Prism (GraphPad) software. Both parametric and non-parametric analyses were performed, depending on sample size. Each experiment was performed at least two times. Details are provided in the figure legends.

## Results

### Early splenic B cells are precursors for both splenic IgM memory B cells and IgM bone ASCs

**Fig 1:**
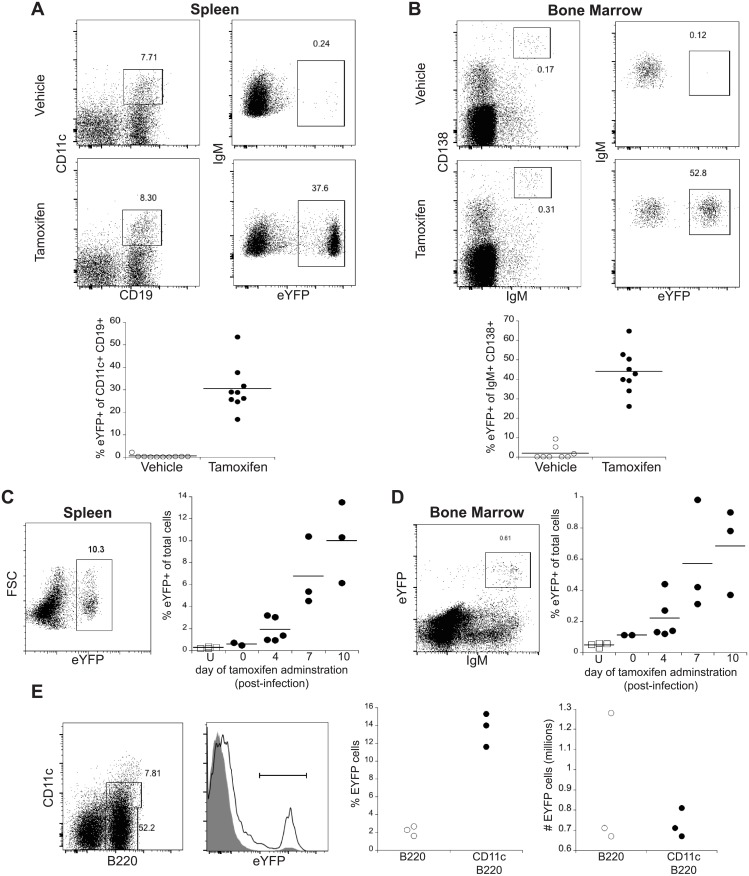
Splenic IgM+ memory B cells and CD138+ IgM+ bone marrow ASCs were derived early following infection. **(A)** Infected (AID-Cre ER^T2^ X eYFP) F_1_ mice were administered tamoxifen, or a vehicle control, on day 10 post-infection, and splenic CD11c-positive CD19+ B cells were analyzed on day 30 post-infection for IgM and eYFP expression by flow cytometry. **(B)** Bone marrow IgM+ B cells from mice treated as in **A** were analyzed for eYFP expression among IgM+ CD138+ cells on 30 days post-infection. Cumulative data from mice analyzed between days 30 and 120 post-infection are shown below the flow cytometry plots in A and B. The differences between the groups in both analyses were statistically significant, as determined using a Mann-Whitney test (p < 0.0001). (**C and D)** Tamoxifen was administered to uninfected (U), or infected (AID-Cre ER^T2^ X eYFP) F_1_ mice, 0, 4, 7, or 10 days post-infection, and spleen (**C)** and bone marrow (**D)** were analyzed for eYFP expression on day 30 post-infection. The frequencies of eYFP+ cells detected in each of the mice are shown in the plots to the right of each dot plot. A multiple comparison ANOVA was used to compare data from uninfected tamoxifen-treated mice, relative to mice that had been treated with tamoxifen on day 0 (p value = 0.99), day 4 (p = 0.61), day 7 (p = 0.0054) and day 10 (p = 0.0002) post-infection. Similar analyses of the bone marrow cells were performed by comparing tamoxifen-treated uninfected mice to mice treated on day 0 (p value = 0.72), day 4 (p = 0.4039), day 7 (p = 0.016), and day 10 (p = 0.0056) post-infection. **(E)** Infected (AID-Cre ER^T2^ X eYFP) F_1_ mice were administered tamoxifen on days 4 and 7 post-infection and were analyzed on day 10 post-infection for eYFP expression in CD11c-negative B220+ (shaded histogram) and CD11c-positive B220+ cells (open histogram), as shown in panels one and two. Cumulative data from the analyses are shown in the plots three and four; the frequencies EYFP+ cells within the B220-positive and the B220/CD11c double-positive populations, indicated by the gate in the second panel, are shown in the third panel (the frequencies were significantly different, as determined using an Unpaired students' T-test (p value = 0.0005). The number of EYFP+ cells within each of the two populations are shown in the fourth panel; the data were not significantly different, as determined using a Mann-Whitney statistical test (p value = 0.10).

Our previous studies showed that the majority of the CD11c-positive IgM memory cells detected on day 30 post-infection expressed somatically-mutated receptors, indicative of AID activity [23]. This observation suggested that IgM memory cell precursors could be identified on the basis of AID expression. Therefore, we utilized AID-Cre-ER^T2^ transgenic mice, described by Dogan et al. [14], to permanently mark IgM memory cells. The strain carries an AID promoter-regulated Cre recombinase whose activity is induced by tamoxifen; it was crossed to a reporter strain that carries a *Gt(Rosa26)Sor*-regulated EYFP allele containing flanking *loxP* sites (B6.Cg-Gt(ROSA)26Sor^tm3(CAG-EYFP)Hze^/J). In B cells that express AID, tamoxifen administration induces Cre recombinase activity that facilitates genetic recombination and subsequent expression of EYFP, thereby permanently marking AID-expressing B cells. We first used the strain to address whether AID-expressing cells induced during acute ehrlichial infection contributed to either long-term IgM population. For these studies, mice were infected, and tamoxifen was administered 10 days later, the peak time of the early CD11c-positive splenic IgM plasmablast response that we described in our previous studies [24]. When the mice were analyzed by flow cytometry on day 30 post-infection, approximately 30% of the CD11c-positive splenic IgM memory cells expressed EYFP (Fig 1a). Although AID is also expressed in GC cells, in our previous study we demonstrated that the IgM memory cells do not express markers characteristic of GC B cells [23]. Similarly, approximately 50% of the CD138-positive IgM-producing bone marrow B cells that we described previously [24] were also labeled following tamoxifen administration on day 10 (Fig 1b). EYFP-positive IgM memory cells and bone marrow ASCs were both identified at least as late as 98 days post-tamoxifen administration. Both the spleen and bone marrow contained cells irreversibly marked following tamoxifen administration as early as day 4 post-infection, indicating that AID expression is induced very early after infection (Fig 1c and 1d). Few, if any, EYFP-labeled B cells were detected in uninfected mice, demonstrating that the EYFP-positive B cells were infection-, and likely, antigen-specific. These studies indicate that early AID-expressing B cells, possibly the early CD11c-positive T-bet+ plasmablasts, give rise to both IgM memory cells and bone marrow IgM ASCs. Not all of the IgM memory cells or plasmablasts expressed EYFP, indicating that not all of the plasmablasts expressed *Aicda* during the window of tamoxifen treatment. Our previous study demonstrated that not all of the IgM memory cells expressed mutated BCRs, so it is also possible that not all of the precursor cells transcribed *Aicda*. To identify *Aicda*-positive cells early during early infection, infected (AID-cre-ER^T2^x EYFP) F_1_ mice were administered tamoxifen on days 4 and 7 post-infection, and splenic B cells were analyzed for EYFP expression three days later, on day 10 post-infection. A higher proportion of the CD11c-positive plasmablasts expressed EYFP (approximately 13%), relative to the CD11c-negative B220+ B cells (approximately 2%; Fig 1e). However, EYFP-positive cells were detected in similar numbers within each population. Although the CD11c-negative B220+ B cells consisted of largely naive follicular B cells, it is possible that some memory precursor B cells were also present. Thus, it is possible that either one or both of the early cell populations give rise to the long-term IgM+ B cells observed on day 30 or later post-infection.

### CD138 IgM bone marrow cells were generated independently of CD4 T cells

**Fig 2:**
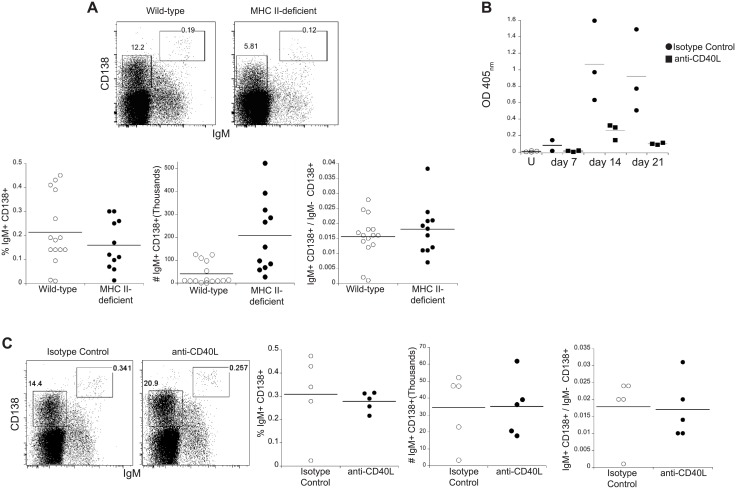
CD138+/IgM+ bone marrow plasmablasts were generated independently of CD4 T cell help. (**A).** Wild-type and MHC class II-deficient mice were infected, and bone marrow was analyzed 30 days post-infection for B cells expressing IgM and CD138. Representative flow cytometry dot plots are shown in the top two panels, indicating the IgM+ plasmablasts (upper right gate in each plot), and IgM-negative plasma cells (gate on left). Cumulative frequencies of IgM+ CD138+ cells are shown in the plot on the bottom left; the differences were not statistically different, as determined using a Mann-Whitney test (p value = 0.32). The number of CD138+ IgM+ cells are shown in the bottom middle plot (p value = 0.0006), and the ratio of IgM+ CD138+ cells to the IgM-negative CD138+ cells are shown on the bottom right plot (p value = 0.63). **(B)** Wild-type mice were immunized with NP(26)-BSA, in alum, and were administered either 200μg anti-CD40L (MR-1) or an irrelevant isotype-matched antibody (2A3) on days 4, 8, and 12 post-immunization. Anti-NP IgG production was analyzed on days 7, 14, and 21 post-infection by ELISA. The anti-CD40L antibody significant inhibited anti-NP responses (p value = 0.0005, as determined using ANOVA). **(C).** Infected wild-type mice were administered 200μg of anti-CD40L, or the isotype-matched irrelevant antibody, on 8, 10, and 12 days post-infection. The cells were analyzed, as in **A,** on 30 days post-infection. The cumulative frequencies of IgM+ CD138+ cells are shown in the third plot (p = 0.42), the number of IgM+ CD138+ cells are shown in the fourth plot (p value = 0.84), and the ratio of IgM+ CD138+ cells to IgM-negative CD138+ cells are shown in the plot on the far right (p value = 0.61); all statistical analyses were performed using a Mann-Whitney test. The data in each of the plots in the figure were compiled from two to three experiments.

It was unknown whether the antigen-specific IgM bone marrow plasmablasts we identified previously [20] were generated in a TI fashion, as has been shown in related studies of bone marrow IgM+ B cells [19, 42]. CD4 T cell help was not required for the generation of IgM bone marrow ASCs in our experimental model, because the ASCs were detected in both wild-type and MHC class II-deficient mice on day 30 post-infection (Fig 2a). The frequency of IgM bone marrow ASCs in MHC class II-deficient mice among total bone marrow cells was modestly lower, relative to wild type mice; moreover, the IgM+ plasmablasts were detected at slightly higher cell numbers in the CD4-deficient mice (Fig 2a, bottom panels). However, there was no difference in the frequency of IgM bone marrow ASCs when measured as a proportion of the frequency of total population of bone marrow CD138+ ASCs within each strain. As an additional test for the requirement for CD4 T cell help in the generation of bone marrow IgM plasmablasts, mice were administered a CD40L-blocking antibody (MR-1), on days 8, 10, and 12 post-infection. In control experiments, this treatment effectively inhibited T cell-dependent responses to adjuvanted NP-BSA (Fig 2b). In contrast, mice treated with the anti-CD40L MAb exhibited very similar frequencies of IgM bone marrow ASCs as control mice (Fig 2c). Our data therefore identify two distinct populations of long-lived cells that are derived early following infection, likely via different mechanisms: a spleen IgM memory population that requires both CD4 T cell help and IL-21 [23], and the CD4 T cell-independent BM ASCs. These data also suggest that B cell fate may be determined in part by access to CD4 T cell help during early infection.

### *Aicda* expression in early splenic plasmablasts requires CD4 T cells, but not TLR9 signals

**Fig 3:**
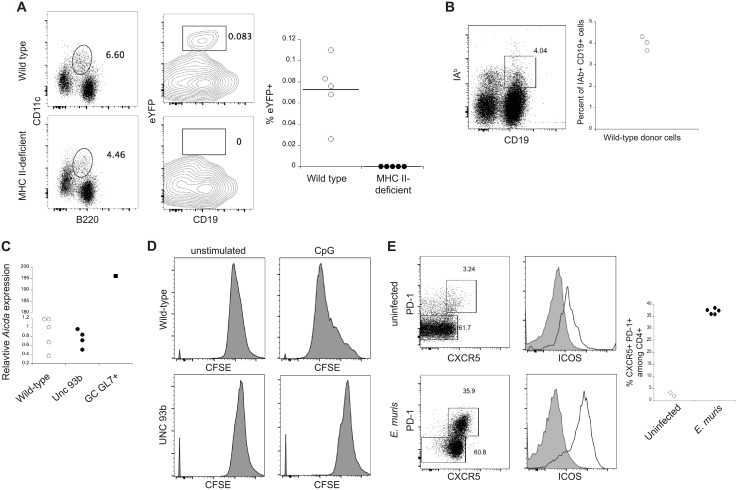
*Aicda* expression in day 10 splenic plasmablasts required CD4 T cells, but did not require TLR9 signaling. **(A)** Magnetic bead-purified naïve splenic B cells from (AID-Cre ER^T2^ X eYFP) F_1_ mice were transferred to wild-type and MHC II-deficient mice; the recipient mice were then infected with *E*. *muris*, and were administered tamoxifen on days 7 and 10 post-infection. The recipient MHC II-deficient mice were treated with 200 μg of a depleting CD4 antibody (GK1.5), immediately following and three days after cell transfer, to eliminate any co-transferred donor T cells. The spleens of recipient mice were analyzed by flow cytometry on day 11 post-infection for CD19- and CD11c-positive donor EYFP+ cells. The percentages of CD11c-positive CD19+ eYFP+ cells detected in wild-type (open circles) and MHC II-deficient mice (closed circles) are shown in the plot on the right (Mann-Whitney test p value = 0.0079). **(B)** Magnetic bead-purified naïve splenic B cells from wild-type mice were transferred to MHC II-deficient mice, and were analyzed as in **A**; the frequency of CD19+ CD11c+ IA^b^+ donor cells among total B cells is shown in the plot on the right. **(C)** CD11c-positive B220+ cells were purified by flow cytometry from the spleens of wild-type (open circles) and UNC93b-deficient (closed circles) mice. mRNA was isolated and RT-PCR was performed to quantify *Aicda* transcripts in each strain. ΔΔCT values were calculated, relative to *β-actin* transcripts. *Aicda* transcripts detected in flow cytometrically-purified GC B cells pooled from six tetanus toxin C fragment-immunized wild-type mice are shown for comparison (closed square). No statistical differences in *Aicda* expression were detected between the wild-type and UNC93b-deficient mice (Mann-Whitney test p value = 0.30). **(D)** T cell-depleted spleens from wild-type and Unc93b-deficient mice were labeled with CFSE and cultured in the presence or absence of CpG (ODN 1826) for three days. CFSE dilution occurred in wild-type, but not UNC93b-deficient mice. **(E)** Spleen cells from uninfected and day 10 post-infection mice were analyzed for CXCR5 and PD-1 surface expression on T_fh_ cells (gated in each dot plot). The gated T_fh_ cells were also analyzed for surface expression of ICOS (open histograms) and compared to CXCR5 PD-1 double-negative cells (closed histogram). The percentages of T_fh_ cells among total CD4+ cells in uninfected (open circles) and *E*. *muris*-infected (closed circles) mice are shown in the plot on the far right (Mann-Whitney test p value = 0.095).

Although we have demonstrated that CD4 T cells are not required for the generation of either the day 10 CD11c-positive plasmablasts [24], nor for the BM plasmablasts, it was possible that T cells were nevertheless required for the induction of AID expression. It is well known that CD4 T cells, in some cases in the context of innate signals, are required for the induction of AID in GC B cells [43, 44]. To identify how AID expression is induced in early IgM plasmablasts, we monitored *Aicda* transcription *in vivo*, using B cells from (AID-cre-ER^T2^x EYFP) F_1_ mice. In these studies, magnetic bead-enriched, T cell-depleted, naïve B cells from (AID-cre-ER^T2^x EYFP) F_1_ mice were transferred to wild-type and MHC II (IA^b^)-deficient mice. The recipient mice were infected with *E*. *muris*, and tamoxifen was administered on days 7 and 10 post-infection. To eliminate any residual donor T cells, the MHC II-deficient recipient mice were administered an anti-CD4 (GK1.5) antibody on day 0 and 3 post-transfer. When the mice were analyzed on day 11 post-infection, EYFP expression in CD11c-positive B220+ plasmablasts was detected in wild-type recipient mice, but not MHC II-deficient mice (Fig 3a), indicating that CD4 T cells were required for *Aicda* expression. Naïve IA^b^-positive donor B cells were detected in MHC II-deficient recipient mice post-infection, indicating that naïve donor B cells were successfully transferred (Fig 3b). The low frequency and number of ehrlichia-specific donor cells was not unexpected, because the frequency of antigen-specific B cells is very low in naïve mice. Thus, even though the day 10 CD11c-postive T-bet+ plasmablasts do not require CD4 T cells for their generation [20], the plasmablasts nevertheless require T cell help to induce *Aicda* mRNA expression. These data also demonstrate that the day 10 CD11c-positive plasmablasts are derived at least in part from naïve splenic precursor B cells.

*Aicda* expression in IgM memory cells required CD4 T cell help; however, it was possible that the *Aicda* expression was also induced by TLR signaling. *E*. *muris* doesn’t express ligands for TLR4, although was possible that the bacteria signal through TLR9, which recognizes bacterial DNA. To address whether TLR signaling was involved, both wild-type and UNC93b-deficient mice (deficient for signaling via TLR3, 6, 7, 8, and 9) were infected with *E*. *muris*. CD11c-positive B220+ plasmablasts were isolated by flow cytometric cell sorting, and qPCR used to detect *Aicda* expression. The UNC93b-deficient CD11c-positive B220+ plasmablasts exhibited *Aicda* expression that was similar to wild-type B cells (Fig 3c). To demonstrate that UNC93b-deficient mice were incapable of responding to TLR9, naïve B cells from wild-type and UNC93b-deficient mice were stimulated with CpGODN1826, and their proliferation was monitored. Wild-type B cells proliferated in response to CpG, but the UNC93b-deficient B cells did not (Fig 3d). The data together indicate that *Aicda* expression likely requires some form of T cell help, but not TLR signaling.

To further investigate the requirement for T cell help, we next investigated whether CD4 T_fh_ cells underwent expansion during early *E*. *muris* infection. We observed a major expansion of CXCR5+ PD-1+ T_fh_ cells on day 10 post-infection, relative to uninfected mice (Fig 3e). Nearly all of the T_fh_ cells exhibited high expression of ICOS, relative to PD-1/CXCR5 double-negative CD4 T cells. Thus, although the day 10 plasmablasts are generated in the absence of CD4 T cells, this occurs in the presence of a vigorous T_fh_ cell response, which is in part required for induction of *Aicda* in the B cells.

### Persistent *Aicda* expression in IgM memory cells

**Fig 4:**
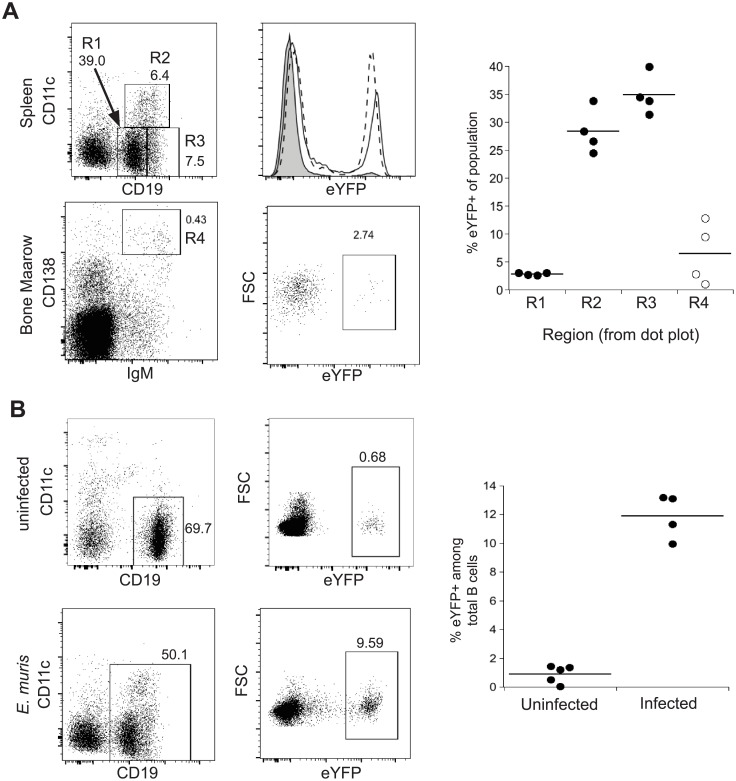
*Aicda* was constitutively expressed in IgM memory B cells. **(A)** (AID-Cre ER^T2^ X eYFP) F_1_ mice that had been infected for at least 30 days, and as long as 200 days post-infection, were administered tamoxifen, and 12 days later the frequencies of EYFP-expressing cells among splenic CD19+ CD11c-negative (Region 1; R1), CD11c- and CD19-double-positive (R2), CD19^hi^ CD11c-negative (R3) B cells, was examined. EYFP expression was also examined in the bone marrow, IgM+ CD138+ B cells (R4; bottom plots). Cumulative data are shown in the plot on the right. The frequency of cells in R2 and R3 both differed significantly from R1 (P<0.0001), as determined by RM one-way ANOVA with Bonferroni's multiple comparison test. (**B)** Naïve and infected (AID-Cre ER^T2^ X eYFP) F_1_ mice were administered tamoxifen and EYFP expression on all B cells was analyzed 12 days later (total B cells were analyzed because CD11c+ IgM memory cells are largely absent in uninfected young mice). Cumulative data is shown in the graph on the right. The data were statistically significant, as determined using a Mann-Whitney test (p value = 0.0007).

We also addressed whether *Aicda* was expressed in long-term IgM memory B cells and bone marrow plasmablasts, by administering tamoxifen to infected (AID-Cre-ER^T2^x EYFP) F_1_ mice on or after day 30 post-infection. Spleen IgM memory cells exhibited EYFP expression 12 days following tamoxifen administration (Fig 4a; approximately 30% of the IgM memory cells were EYFP-positive). In contrast, lower percentages of EYFP+ cells were detected among the bone marrow plasmablasts (R4 in the bottom panels of Fig 4a). We also detected EYFP expression in a previously unidentified population of splenic CD19^hi^ CD11c-negative B cells (R3 in Fig 4a); other studies have indicated that these B cells, which were not resolved in previous studies, are phenotypically similar to CD11c-positive IgM memory cells we have characterized (unpublished data). Although we cannot completely exclude the possibility that some memory cells had recently emigrated from GCs, where they expressed AID, it is unlikely that 30% of the cells had emigrated during the time tamoxifen was administered. These data indicate that *Aicda*-expression can be maintained in IgM memory cells during low-level chronic ehrlichial infection, in the absence of class switching. To rule out any possibility that the (AID-Cre-ER^T2^x EYFP) F_1_ mice can express the Cre recombinase spontaneously, naïve mice were administered tamoxifen and EYFP expression on B cells was examined 12 days later. Naïve B cells from (AID-Cre-ER^T2^x EYFP) F_1_ mice exhibited low expression of EYFP, relative to B cells from infected (AID-Cre-ER^T2^x EYFP) F_1_ mice (Fig 4b). EYFP expression among CD19+ B cells was significantly higher in infected mice that had established the IgM memory population, compared to naïve mice. These data demonstrate that CD11c-positive IgM memory cells can maintain transcription of *Aicda*, apparently indefinitely; whether AID is produced and is functional, and the impact of possible AID function for the maintenance of long-term IgM memory, is not yet known.

## Discussion

Our findings reveal that both long-term antigen-specific IgM memory cells and bone marrow plasmablasts are derived from precursor cells present early during ehrlichial infection, well before the generation of canonical class-switched memory B cells that develop in GCs [45, 46]. Our studies therefore highlight what is likely a novel pathway of memory development that is important for the generation and maintenance of long-term unswitched CD11c- T-bet-positive memory B cells. Although we have demonstrated that the long-term IgM-positive B cells in our model are derived early during infection, as early as day 4 post-infection, we have not yet resolved whether the memory cells and plasmablasts are generated independently, or via a single pathway. Given their phenotypic similarities, it is possible that both long-lived B cell populations are derived from the early splenic CD11c- T-bet-positive plasmablasts that we have described [24]. Indeed, nearly all of the ehrlichial-specific IgM detected on day 10 post-infection was secreted by the early CD11c-positive plasmablasts, not CD11c-negative B cells [24, 47]. However, plasmablasts are by definition short-lived cells, so an alternative explanation is that the long-term IgM-positive memory cells are derived independently, perhaps from CD11c-negative or CD138-negative follicular B cells. Although EYFP-positive cells were found at much higher frequencies among CD11c-positive plasmablasts, EYFP+ CD11c-negative B cells were found in similar numbers as the CD11c-positive plasmablasts. Ongoing studies will help to resolve the origin(s) of the long-term IgM B cells. These findings nevertheless highlight a novel pathway for the generation of long-term IgM memory and antibody-secreting plasmablasts in the context of a TI response to infection. Our and others' work highlight how infections can induce B cell responses that differ from those elicited by canonical non-infectious antigens, often challenging established dogma.

Although the day 10 CD11c- T-bet-positive plasmablasts do not require CD4 T cell help for their generation, the signals required for the subsequent differentiation of the plasmablasts to either IgM B cell memory cells or BM ASCs are not yet known. However, the IgM memory cells require CD4 T cells (and IL-21) for their generation [23], whereas the BM plasmablasts do not, suggesting that the availability of T cell help may be a key factor in what drives the development of B cell memory population versus long-lived BM plasmablasts. In this regard, we have observed a large population of splenic T_fh_ cells that are present at the time of the early CD11c-positive plasmablast response. Given the magnitude of the T_fh_ cell response, the data suggest that T cell help is not limited by cell number, and it is possible that the fate of the B cells may be determined instead by their physical location. In such a model, extrafollicular B cells that fail to interact with T_fh_ cells may exit the spleen and migrate to the bone marrow, whereas B cells that enter follicles may elicit T cell signals that drive IgM memory cell development. Both populations likely differentiate independently of GCs, which are suppressed during early ehrlichial infection [48]. Although we have not yet formally resolved the requirements for GCs in our experimental model, other studies of non-canonical memory B cells suggest IgM memory cells follow a GC-independent pathway [4, 5, 49]. Alternately, affinity may be a factor in the fate decision; higher affinity B cells may elicit more T cell help, thereby promoting IgM memory B cell development, whereas cells that fail to elicit sufficient T cell help may follow a default pathway to become bone marrow plasmablasts. Finally, it is likely that the IgM memory cells are driven by T_fh_ cells that produce IFNɣ, as has been observed during *Salmonella typhi* infection [50]. Indeed, IFNɣ is likely responsible for inducing T-bet expression in the CD11c+ plasmablasts we have described previously [24, 51].

Our demonstration that the bone marrow IgM plasmablasts are generated independently of T cells is consistent with other studies that have shown that TI responses can elicit long-term antibody production and/or protection [21, 22, 42]. Those studies demonstrated that long-term IgM production could be maintained indefinitely, via two different mechanisms. One possibility is that IgM is maintained by the continual recruitment of short-lived plasma cells [21, 22]. Alternatively, it has been proposed that TI pathogens can induce long-lived plasma cells [42]. *E*. *muris* establishes a low-level chronic infection, although it is unknown if sufficient antigen can be derived from these intracellular pathogens to maintain IgM plasmablasts via antigen stimulation in the bone marrow. Alternatively, low level inflammation in the bone marrow may drive the production of factors that support plasmablast/plasma cell maintenance [52].

Our studies also allowed us to address the source of the signals that drive *Aicda* expression in early splenic CD11c- T-bet-positive plasmablasts. Although these cells are generated in the absence of CD4 T cell help, we show that CD4 T cell signals are nevertheless required to induce *Aicda* transcription. We propose *Aicda* transcription is driven by interactions with T_fh_ cells that are abundant in the spleen at that time, perhaps via classical CD40:CD40L interactions. Unlike what has been described in other studies [44, 53–55], *Aicda* transcription did not require TLR9, and unlikely involves other TLRs, because the ehrlichiae lack classical TLR ligands. Other innate signals may substitute during ehrlichial infections, although the nature of these signals and/or receptors is currently unknown. The observation that *Aicda* expression was maintained, apparently indefinitely, in the IgM memory B cell population, suggests that the same or different factors that elicit *Aicda* expression on day 10 post-infection are maintained in the IgM memory cells, perhaps as a consequence of low-level inflammation. The consequences of long-term AID expression in IgM memory B cells is unknown, but it has been suggested that chronic low-level AID expression in memory B cells can promote polyreactivity, self-reactivity, and clonal elimination [56].

We also resolved a previously unidentified CD19^hi^ CD11c-negative B cell population, on the basis that this these B cells also expressed AID (i.e., they were found to be EYFP-positive following tamoxifen administration). It is likely that this CD19^hi^ CD11c-negative population is closely related to the IgM memory cells we have described previously, and will undergo further analysis.

Our studies also shed light on the origin and function of CD11c- T-bet-positive B cells, which are now emerging as an important B cell subset involved in both host defense and autoimmunity. Although it is unclear whether CD11c and/or T-bet define a single or multiple functionally distinct B cell subsets, our previous and current findings support the hypothesis that such B cells include IgM memory B cells [23]. Although some CD11c- T-bet-positive B cells are generated in response to TLR signaling [36, 38], our data indicate that these signals are not required, although other innate signals likely substitute for TLRs. T-bet activity may also be responsible for maintaining persistent *Aicda* expression.

Our studies highlight a novel pathway for the development of both IgM memory cells and long-term bone marrow plasmablasts. We have shown that IgM production is maintained indefinitely following *E*. *muris* infection, and it is likely that both non-switched populations are important for maintenance of humoral memory. It will be important to address whether similar mechanisms contribute to long-term immunity in humans after either infection or vaccination.

## Supporting information

S1 File ARRIVE checklist. (PDF)
